# Spinal and Paraspinal Malignant Peripheral Nerve Sheath Tumors (MPNSTs): Survival, Local Recurrence, and the Relative Importance of Resection Extent and Margin Status

**DOI:** 10.1177/21925682261465343

**Published:** 2026-07-02

**Authors:** Leevi Toivonen, Anton Denisov

**Affiliations:** 1 Department of Orthopedics and Traumatology, 60670 Tampere University Hospital and Tampere University, Tampere, Finland; 2 Department of Orthopedics and Traumatology, Mollet University Hospital, Barcelona, Spain; 3 Research and biostatistics division, The Taylor Collaboration, San Francisco, CA, USA

**Keywords:** malignant peripheral nerve sheath tumor, MPNST, malignant triton tumor, MTT, spinal, paraspinal, overall survival, local recurrence free survival

## Abstract

**Study Design:**

Systematic literature review.

**Objectives:**

To characterize clinical features, treatment patterns, and outcomes of spinal and paraspinal malignant peripheral nerve sheath tumors (MPNSTs) and to evaluate the relative prognostic importance of extent of resection and surgical margin status.

**Methods:**

A search of published cases of spinal and paraspinal MPNSTs with individual-level treatment and outcome data was performed. Overall survival (OS) and local recurrence-free survival (LRFS) were analyzed using Kaplan–Meier estimates and multivariable Cox regression. Competing-risk regression was used to assess local recurrence while accounting for death as a competing event. Model performance was compared using the Akaike information criterion.

**Results:**

Among 230 included patients, median OS was approximately 15 months, and median RFS was approximately 25 months, with a high early local recurrence rate. Metastatic disease at presentation was the strongest predictor of death (HR 3.02, 95% CI 1.57–5.84). Gross total resection was independently associated with improved OS (HR 0.37, 95% CI 0.23–0.58) and LRFS (HR 0.38, 95% CI 0.20–0.72) and demonstrated superior model performance over surgical margin status. In a competing-risk analysis, intralesional resection was independently associated with increased local recurrence (sub-distribution HR 1.78, 95% CI 1.09–2.92), while neurofibromatosis type 1 also conferred elevated local recurrence risk.

**Conclusions:**

Spinal and paraspinal MPNSTs are characterized by poor survival and high early local recurrence. Extent of resection is the dominant surgical determinant of OS, whereas margin status primarily influences LRFS. These findings support the hypothesis that macroscopic total resection should be pursued even when negative margins cannot be achieved.

## Introduction

Malignant peripheral nerve sheath tumors (MPNSTs) are a rare, aggressive type of soft tissue sarcomas.^1,2^ Over 50% of MPNSTs arise in individuals with neurofibromatosis type 1 (NF1).^3^ Benign schwannomas occur in excess in patients with neurofibromatosis 2 (NF2), but their malignant transformation is extremely rare.^4^ Prior radiation exposure has been linked to one-tenth of cases,^2^ while the remaining MPNSTs arise sporadically. Total removal with wide margins is considered the treatment of choice for MPNSTs.^5,6^ Tumor location in the spine may preclude achieving marginal resections, further worsening the prognosis of axial MPNSTs.^7^Five-year survival rates between 16% and 63% have been reported with spinal and paraspinal MPNSTs.^3,7,8^ Historically, NF1 status has been associated with impaired prognosis, but this discrepancy appears to be diminishing in more recent data.^9,10^ A Malignant Triton tumor (MTT), a subtype of MPNST with rhabdomyoblastic differentiation, is generally characterized by a higher degree of histological malignancy and thus has an even more dismal prognosis.^11^

Owing to the rarity of spinal MPNSTs, the related literature is limited to case reports and small case series. Chou et al. reported the largest case series to date (n=29), using data from an international multicenter database.^6^ They could not demonstrate improved local or overall survival with Enneking-appropriate (clear margin) resections. This makes decisions about extensive surgeries with high morbidity and unclear benefits challenging. Radiotherapy appears to be of limited value, although it is oftentimes administered, especially for residual tumors and local recurrences.^3,8^ Chemotherapy is primarily reserved for metastatic disease and provides minimal benefit.^8^

The objective of this study was to conduct a literature review on all reported cases of MPNST at intraspinal or paraspinal locations. Based on the reported data, the aim was to synthesize prognostic factors to help guide treatment.

## Methods

### Study Design and Literature Search

This study was conducted as a systematic review with an individual patient data meta-analysis of reported cases of spinal and paraspinal (resection margin touching the spine) MPNSTs. The review protocol followed PRISMA guidelines.^12^

A comprehensive search of PubMed (MEDLINE), Web of Science, and Scopus databases was performed from inception through December 2024 using a combination of keywords related to MPNST and spinal and paraspinal anatomy. Only English-language articles providing individual-level clinical, treatment, and outcome data were eligible.

Titles and abstracts were screened independently, followed by full-text review of potentially eligible articles. Inclusion required histologically confirmed MPNST or MTT involving the spinal canal, vertebral column, or paraspinal region, with extractable patient-level data on treatment and survival or recurrence.

**Figure 1.**
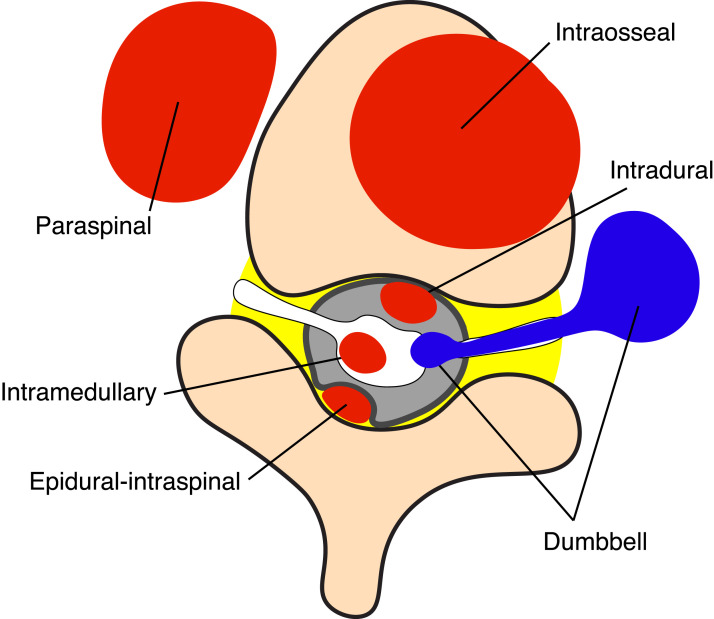
Tumor classification based on morphology

Tumors were categorized by anatomic region (cervical, thoracic, lumbar, sacral, or multilevel) and morphology (intramedullary, intradural, extradural-intraspinal, dumbbell, intraosseous, or paraspinal) based on imaging descriptions and operative reports (Figure 1).

### Data Extraction

From each eligible report, the following variables were extracted when available: age, sex, NF1 or NF2 status, prior radiotherapy, histologic subtype (MPNST or MTT), tumor location and morphology, presence of metastases at presentation, extent of resection (gross total or subtotal), surgical margin (marginal/wide or intralesional), use of initial radiotherapy and chemotherapy, recurrence or progression, and survival outcomes.

Extent of resection was defined as gross total resection (GTR) when complete macroscopic removal was reported and subtotal resection (STR) for all other resections. Margins were dichotomized as marginal or intralesional based on operative and pathological descriptions.

### Outcomes

Overall survival (OS) was defined as the time from diagnosis or initial surgery to death or last follow-up. Local recurrence-free survival (LRFS) was defined as the time from surgery to the first local recurrence, local progression following STR, or last follow-up. Distant recurrence was not analyzed separately because data were limited and inconsistently reported. For competing-risk analyses, local recurrence was treated as the event of interest and death before local recurrence was treated as a competing event.

### Statistical Analysis

Continuous variables are presented as the mean ± standard deviation or median (interquartile range), and categorical variables are presented as counts and percentages. Survival curves were estimated using the Kaplan–Meier method and compared using log-rank testing. OS was analyzed using multivariable Cox proportional hazards regression. Histologic subtype violated the proportional hazards assumptions and was therefore incorporated as a stratification variable (MPNST or MTT). Covariates included age, NF1 status, metastatic disease at presentation, initial radiotherapy, and chemotherapy.

To assess the relative prognostic value of surgical strategy, separate models were constructed that included (1) extent of resection alone and (2) surgical margin status alone, owing to strong collinearity between these variables (with marginal margins occurring exclusively after GTR), precluding reliable simultaneous estimation of independent effects. Model performance was compared using the Akaike information criterion (AIC). LRFS was evaluated using Cox regression, following the same modeling strategy.

Competing-risk regression, following the Fine–Gray model, was used to analyze local recurrence while accounting for death as a competing event and adjusting for NF1 status, metastatic disease at presentation, initial radiotherapy, and chemotherapy. Hazard ratios (HRs) and sub-distribution hazard ratios (sHRs) are reported with 95% confidence intervals. Statistical analyses were performed using R (RStudio v2025.05.0+496). A two-sided p-value of less than 0.05 was considered statistically significant. Patients with missing data were excluded from analyses requiring those variables. No imputation was performed.

## Results

### Key Findings

Based on published reports, spinal and paraspinal MPNST are characterized by poor OS (median 15 months) and LRFS (median 25 months) with high early mortality and risk of local recurrence. GTR was independently associated with improved OS (HR 0.37, 95% CI 0.23–0.58) and LRFS (HR 0.38, 95% CI 0.20–0.72).

### Study Selection

**Figure 2.**
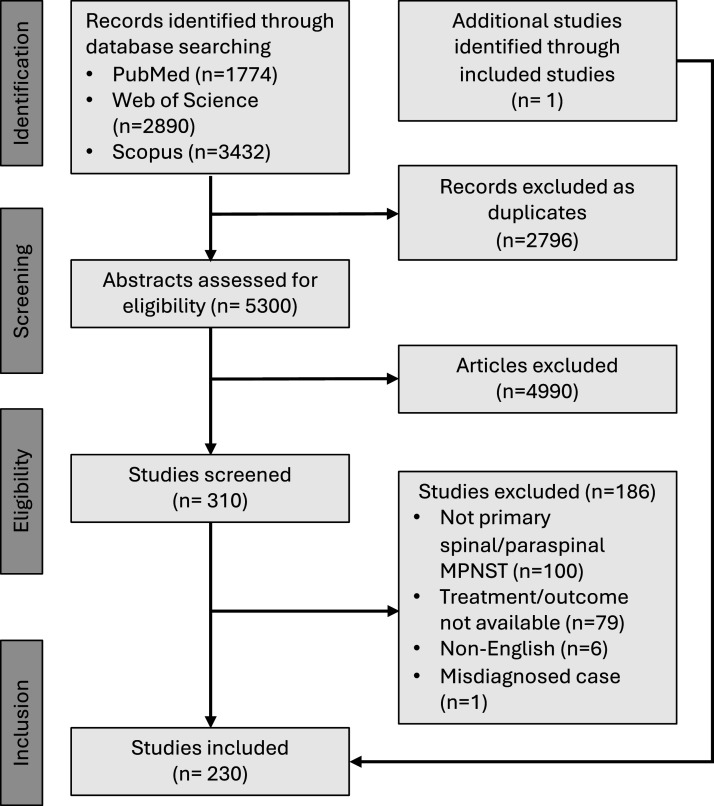
PRISMA flow diagram

The initial search yielded 8096 articles. After automatic and manual duplicate removal, 5300 articles remained for title and abstract screening. Thereafter, 310 articles underwent full text screening, of which 125 articles satisfied the inclusion criteria (Table 1). One additional eligible study was identified from the references of the included articles. A flowchart of the screening process is presented in Figure 2.

**Table 1.** List of the Included Studies

Author	Year	Country	N
Abdallah et al.^13^	2024	Jordan	1
Acharya et al.^14^	2001	India	1
Adamson et al.^15^	2004	USA	2
Aponte-Caballero et al.^16^	2024	Colombia	1
Asano et al.^17^	2016	Japan	1
Baharvahdat et al.^18^	2018	Iran	1
Berner et al.^19^	2021	USA	1
Bhoir et al.^20^	2012	India	1
Bouty et al.^21^	2018	France	1
Burks et al.^22^	2017	USA	1
Cano et al.^23^	2006	Spain	1
Celli et al.^24^	1995	Italy	6
Chamoun et al.^25^	2009	USA	1
Chander et al.^26^	2004	USA	1
Chandler et al.^27^	1994	UK	3
Chao et al.^28^	2008	USA	1
Chen et al.^29^	2019	China	8
Chen et al.^30^	2024	China	1
Chowdhury et al.^31^	2023	USA	4
Christi et al.^32^	2021	Indonesia	1
Crandon et al.^33^	1995	Jamaica	1
Dartnell et al.^34^	2009	UK	1
Dawes et al.^35^	2014	Australia	1
De Jong et al.^36^	1990	Netherlands	1
Devadoss et al.^37^	2014	India	1
Dimou et al.^38^	2009	Australia	1
Falavigna et al.^39^	2016	Brazil	1
Farrokhi et al.^40^	2023	Iran	4
Fauth et al.^41^	2009	Austria	1
Fisher et al.^42^	1994	UK	2
Fletcher et al.^43^	1987	UK	1
Foley et al.^44^	1980	USA	7
Frijns et al.^45^	1989	Netherlands	1
Ghailane et al.^46^	2019	France	1
Ghosh et al.^47^	2011	India	1
Gilder et al.^48^	2018	USA	1
Gnanalingham et al.^49^	2004	UK	1
Gong et al.^50^	2018	China	14
Hagi et al.^51^	2016	Japan	1
Hara et al.^52^	2008	Japan	1
Honda et al.^53^	2020	Japan	3
Hénaux et al.^54^	2011	France	1
Ido et al.^55^	1998	Japan	1
Imazu et al.^56^	2006	Japan	1
Iqbal et al.^57^	2009	USA	1
Isla et al.^58^	2000	Spain	1
Isler et al.^59^	1996	USA	1
Jaing et al.^60^	2015	Taiwan	1
James et al.^61^	2008	UK	2
Karn et al.^62^	2024	Nepal	1
Kett-White et al.^63^	2000	UK	1
Khan et al.^64^	1998	Pakistan	1
Kiatisevi et al.^65^	2014	Thailand	1
Kim et al.^66^	2008	Korea	1
Kim et al.^67^	2021	Korea	1
Klimo et al.^68^	2009	USA	2
Kourea et al.^7^	1998	USA	25
Krepler et al.^69^	2002	Austria	1
Lai et al.^70^	2006	Taiwan	1
Larson et al.^71^	2022	USA	1
Lau et al.^72^	2014	USA	1
Lee et al.^73^	1975	USA	1
Lee et al.^74^	2010	Korea	1
Li et al.^75^	2014	China	1
Lin et al.^76^	2009	Taiwan	1
Linder et al.^77^	2019	UK	1
Liu et al.^78^	2019	China	1
Matsumoto et al.^79^	2018	Japan	13
Mazel et al.^80^	2002	France	3
McLaughlin et al.^81^	2011	USA	1
Meshikhes et al.^82^	2016	Saudi Arabia	1
Miyakoshi et al.^83^	2007	Japan	1
Moon et al.^84^	2008	Korea	1
Mortelé et al.^85^	1998	Belgium	1
Murovic et al.^86^	2010	USA	1
Mut et al.^87^	2004	Turkey	1
Nanda et al.^88^	2015	USA	2
Navarro et al.^89^	2000	Canada	2
Noreña-Rengifo et al.^90^	2021	Colombia	1
Ogose et al.^91^	2001	Japan	2
Ordóñez^92^	1998	USA	1
Palejwala et al.^93^	2017	USA	1
Paolini et al.^94^	2006	Italy	1
Paparella et al.^95^	2022	Italy	1
Park et al.^96^	2013	Korea	1
Park et al.^97^	2022	USA	2
Patil et al.^98^	2015	India	1
Peng et al.^99^	2011	China	1
Peterson et al.^100^	2018	USA	1
Posma et al.^101^	2003	Netherlands	1
Pratap et al.^102^	2007	Nepal	1
Prieto et al.^11^	2012	Spain	1
Qi et al.^103^	2023	China	1
Roopesh Kumar et al.^104^	2014	India	1
Rosette et al.^105^	1960	USA	1
Sasamori et al^106^.	2012	Japan	1
Seppälä et al.^107^	1993	Finland	6
Sharma et al.^108^	1989	India	1
Sodhi et al.^109^	2014	India	1
Stark et al.^110^	2001	Germany	1
Sundaram et al.^111^	2018	India	1
Suzuki et al.^112^	2014	Japan	1
Tan et al.^113^	2011	Australia	1
Thomas et al.^114^	2014	USA	1
Topal et al.^115^	2004	Turkey	1
Ugwu et al.^116126^	2017	Nigeria	1
Valdueza et al.^117^	1991	Germany	5
Wang et al.^118^	1985	China	2
Wang et al.^119^	2018	China	1
Wang et al.^120^	2020	China	1
Watier et al.^121^	2000	France	1
West et al.^122^	1997	USA	1
Woodruff^123^	1976	USA	2
Woodruff et al.^124^	1993	USA	1
Woodruff et al.^125^	1994	Australia	1
Wu et al.^126^	2014	China	1
Wu et al.^127^	2020	USA	1
Xu et al.^128^	2012	China	1
Yan et al.^129^	2012	China	1
Yang et al.^130^	2008	China	1
Yi et al.^131^	2023	China	1
Ylmaz et al.^132^	2004	Turkey	1
Yone et al.^133^	2004	Japan	1
Zang et al.^134^	2015	China	1
Zhu et al.^135^	2012	China	8

### Summary of the Included Studies

In total, 126 articles were included from 29 countries, with the United States (n = 29, 23.0%), China (n = 17, 13.5%), and Japan (n = 12, 9.5%) being the most represented. Publication years spanned 1960 to 2024. Most studies (81%) reported one case, whereas three articles included more than 10 eligible cases.

### Summary of the Patient Cases

Studies reported 230 cases (56% men, mean age 37.5 [SD, 17.7] years) (Table 2). One-third were associated with NF1, and three cases were reported in association with NF2.^77,107,126^NF1-associated cases were younger (mean age 30.2 [SD, 14.8] years) than non-NF1 cases (38.4 [SD, 18.7] years) and those with unknown NF status (43.4 [SD, 16.4] years) (p = 0.003). One-fifth of cases had received prior radiotherapy with a median latency from irradiation to MPNST diagnosis of 12.0 years (IQR, 8.4–17.8 years).

**Table 2.** Basic Characteristics of Patients, Tumors, and Treatment per Tumor Histology

​	MPNST		MTT	
	Mean	SD	Mean	SD
Age, years	38	18	32	17
​	**Count**	**Percentage**	**Count**	**Percentage**
Sex				
Male	116	54.7	13	76.5
Female	96	45.3	4	23.5
NR	1	​	-	​
Neurofibromatosis status				
NF-1	55	34.2	7	46.7
NF-2	3	1.9	-	​
None	103	64.0	8	53.3
NR	52	​	​	​
Prior irradiation				
Yes	28	21.2	3	18.8
No	104	78.8	13	81.3
NR	81	​	1	​
Anatomic region				
Cervical	50	23.9	4	23.5
Thoracic	50	26.9	5	29.4
Lumbar	55	29.6	6	35.3
Sacral	17	9.1	1	5.9
Multiple	14	7.5	1	5.9
NR	27	​	-	​
Morphology				
Intramedullary	4	2.1	-	​
Intradural	45	23.2	2	11.8
Extradural-intraspinal	12	6.2	-	​
Dumbbell	55	28.4	7	41.2
Intraosseous	19	9.8	1	5.9
Paraspinal	59	30.4	7	41.2
NR	19	​	-	​
Metastases at presentation				
No	147	89.1	12	100
No, but multifocal tumor	1	0.6	-	​
Yes	17	10.3	-	​
NR	48	​	5	​
Resection				
No	15	7.0	1	5.9
Subtotal resection	86	40.4	4	23.5
Gross total resection	109	51.2	12	70.6
Margins				
Intralesional	135	73.0	12	75.0
Marginal	46	24.9	4	25.0
Marginal following intralesional resection	4	2.2	-	​
NR	28	​	1	​
Radiation therapy				
No	100	51.8	6	37.5
Initial radiotherapy (as part of initial treatment)	79	40.9	9	56.3
After recurrence	14	7.3	1	6.3
NR	20	​	1	​
Chemotherapy				
Yes	52	26.8	8	47.1
No	145	73.2	9	52.9
NR	15	​	-	​

MPNST, malignant peripheral nerve sheath tumor; MTT, malignant triton tumor; SD, standard deviation; NR, not reported.

Tumor histology was MTT in 17 cases (7.4%), whereas the remaining cases were classified as MPNST. Tumors were evenly distributed across the spine (cervical, thoracic, and lumbar locations; 27%, 27%, and 30%, respectively). Paraspinal (n = 66, 31%) and dumbbell (n = 63, 30%) morphology were most common, followed by the intradural type (n = 47,22%).

Resection was performed in 92% of cases, with GTRs outnumbering STRs (121 vs. 90). In 50 cases (25%), the index resection could be interpreted as marginal, whereas 73% of resections remained intralesional. Resection was supplemented with adjuvant radiotherapy (initial radiotherapy) in 82 cases, more often after STR than after GTR (54.9% vs. 45.1%; p = 0.029). Following recurrence, radiotherapy was administered to 15 patients (7%). Resection was complemented by chemotherapy in 47 patients, independent of the extent of resection (53.2% after STR vs. 46.8% after GTR; p = 0.119). In addition, chemotherapy was administered for disease progression in eight cases.^26,41,45,49,91,95,96^ Of the 19 patients who did not receive primary surgery, six underwent radiotherapy,^7,44,51,79,98^ and six underwent chemotherapy.^7,30,36,71,77,79^ In one case, the patient first refused surgery, which was performed later with advanced disease.^39^

### Overall Survival

**Figure 3.**
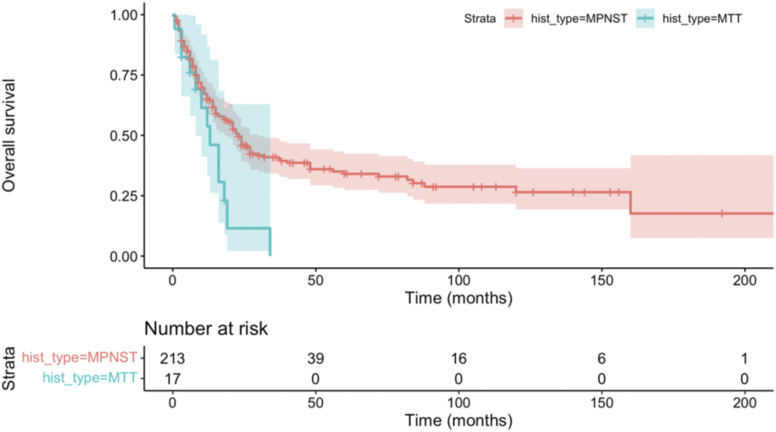
Estimated overall survival (OS) for the overall cohort stratified by histologic subtype. Shaded areas represent the 95% confidence intervals, and tick marks indicate censored observations. The number of patients at risk at predefined timepoints is presented below the plot

**Figure 4.**
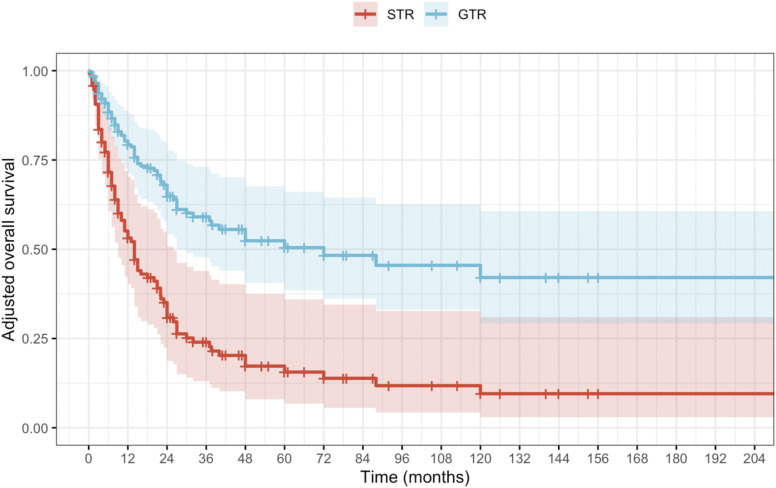
Estimated adjusted overall survival (OS) of patients who underwent gross total resection (GTR) compared with those who underwent subtotal resection (STR). Survival curves represent model-based estimates derived from a Cox proportional hazards model, with covariates fixed at their median (continuous variables) or modal (categorical variables) values; therefore, no number-at-risk table is presented

The estimated median OS for the overall cohort was approximately 15 months, with MTT histology being associated with increased mortality (Figure 3). Patients who underwent GTR demonstrated markedly superior survival rates compared with those who received STR (Figure 4). Likewise, negative margin status clearly conveyed superior OS probabilities (Supplementary material, Figure S1).

In multivariable Cox regression stratified by histologic subtype, metastatic disease at presentation emerged as the strongest adverse prognostic factor (HR 3.02, 95% CI 1.57–5.84; p < 0.001) (Table 3). Extent of resection was independently associated with survival, with STR conferring a significantly higher hazard of death than GTR (HR 2.71, 95% CI 1.72–4.28; p < 0.001). Initial radiotherapy was associated with improved OS (HR 0.62, 95% CI 0.40–0.96; p = 0.03), whereas age and NF1 status were not statistically significant.

**Table 3.** Multivariable Cox Proportional Hazards Model for Overall Survival, Stratified by Histologic Subtype

Variable	HR (95% CI)	P
Age (per year)	1 (0.99–1.01)	0.673
Metastases at diagnosis (yes vs no)	3.02 (1.57–5.84)	<0.001
NF1 (yes vs no)	1.43 (0.93–2.19)	0.104
Extent of resection (STR vs GTR)	2.71 (1.72–4.28)	<0.001
Initial radiotherapy (yes vs no)	0.62 (0.4–0.96)	0.03

HR, Hazard ratio; CI, Confidence interval; NF1, neurofibromatosis type 1; STR, subtotal resection; GTR, gross total resection.

Model comparison using AIC demonstrated superior performance of the extent-of-resection model compared with models incorporating margin status alone or in combination, supporting extent of resection as the dominant surgical determinant of OS (Supplementary material, Table S1).

### Local Recurrence-free Survival

**Figure 5.**
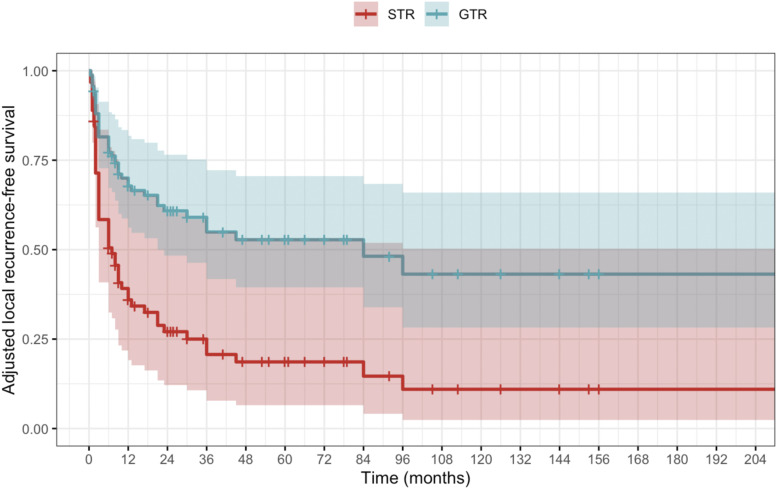
Estimated local recurrence-free survival (LRFS) for patients who underwent gross total resection (GTR) exceeds that for those who underwent subtotal resection (STR). Survival curves represent model-based estimates derived from a Cox proportional hazards model, with covariates fixed at their median (continuous variables) or modal (categorical variables) values; therefore, no number-at-risk table is presented

Among patients with available local recurrence data, the median LRFS was approximately 25 months, characterized by a steep early decline followed by a plateau in longer-term survivors (Supplementary material, Figure S2). GTR conferred a clear advantage for both early and durable disease control (Figure 5).

Marginal margins occurred exclusively following GTR, demonstrating complete separation and strong collinearity between margin status and extent of resection (Fisher’s exact test, p < 0.001) (Supplementary material, Table S2).

In multivariable Cox analysis, MTT histology was strongly associated with worse LRFS (HR 4.46, 95% CI 1.97–10.06; p < 0.001) (Table 4). STR was independently associated with increased recurrence risk (HR 2.6; 95% CI 1.38–5.02; p = 0.003). Age, initial radiotherapy, and chemotherapy were not significant predictors in this model. As with OS, AIC comparison favored models incorporating extent of resection over those based on margin status (Supplementary material, Table S3).

**Table 4.** Multivariable Cox Regression for Local Recurrence–free Survival (LRFS), Model 1

Variable	HR	95% CI	p-value
Age (per year)	0.99	0.98–1.01	0.41
Histology (MTT vs MPNST)	4.46	1.97–10.06	<0.001
Initial radiotherapy (yes vs no)	0.83	0.46–1.51	0.54
Initial chemotherapy (yes vs no)	1.01	0.50–2.02	0.98
Extent of resection (STR vs GTR)	2.63	1.38–5.02	0.003

HR, hazard ratio; CI, confidence interval; MTT, malignant triton tumor; MPNST, malignant peripheral nerve sheath tumor; STR, subtotal resection; GTR, gross total resection.

A trend toward better survival probabilities was observed with more caudal tumor locations (Supplementary material, Table S4). Intramedullary tumors conveyed inferior survival when compared with other tumor morphologies.

### Competing-Risk Analysis for Local Recurrence

**Figure 6.**
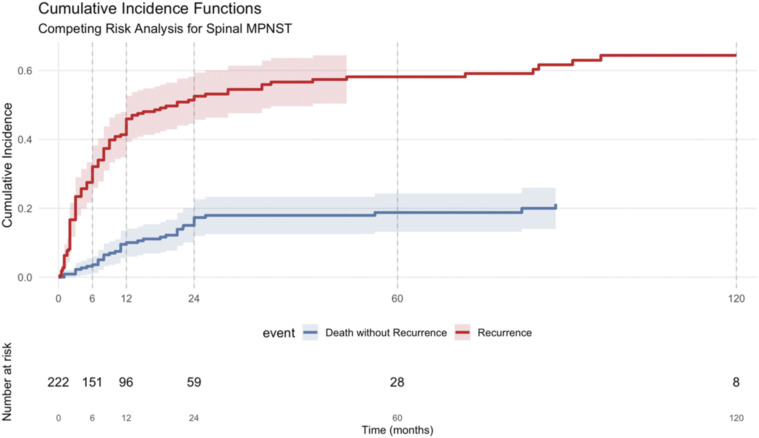
Cumulative incidence functions for local recurrence and death before recurrence in patients with spinal malignant peripheral nerve sheath tumors. Recurrence was treated as the event of interest, and death before recurrence as a competing event. Shaded areas represent 95% confidence intervals. The number of patients at risk at predefined timepoints is presented below the plot

Competing-risk analysis demonstrated a high early cumulative incidence of local recurrence, reaching approximately 55% at 24 months (Figure 6). Death prior to local recurrence occurred less frequently, with a cumulative incidence of approximately 18% at 24 months. NF1 patients demonstrated a higher local recurrence trend than non-NF1 patients, although this did not reach statistical significance (Gray’s test, p = 0.063).

**Figure 7.**
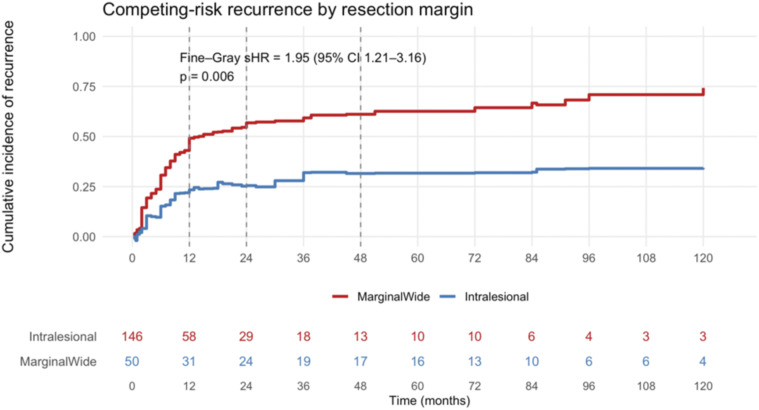
Adjusted cumulative incidence of local recurrence stratified by surgical margin status, treating death as a competing event and adjusted for neurofibromatosis type 1, metastatic disease at presentation, initial radiotherapy, and initial chemotherapy. The number of patients at risk at predefined timepoints is presented below the plot

Intralesional resection was independently associated with increased local recurrence (Fine–Gray sub-distribution hazard ratio 1.95, 95% CI 1.21–3.16; p = 0.006) (Figure 7). At 12 months, the adjusted cumulative incidence of local recurrence was 49.2% (95% CI 39.4–60.0) following intralesional resection compared with 23.4% (95% CI 11.1–36.8) after marginal excision, corresponding to an absolute difference of 25.8%. By 24 months, local recurrence increased to 56.8% (95% CI 47.1–67.8) versus 25.5% (95% CI 11.7–40.6), respectively, corresponding to an absolute difference of 31.3%. Bootstrap-derived confidence intervals confirmed the robustness of these estimates.

In the multivariable Fine–Gray competing-risk regression, intralesional margins were independently associated with an increased risk of local recurrence (sHR 2.08, 95% CI 1.20–3.63; p = 0.010), as was NF1 (sHR 1.64, 95% CI 1.04–2.61; p = 0.034) (Table 5). Metastases at presentation were not significantly associated with local recurrence after accounting for competing mortality (sHR 1.85, 95% CI 0.72–4.78; p = 0.200). Initial radiotherapy showed a borderline association with reduced local recurrence risk (sHR 0.62, 95% CI 0.38–1.00; p = 0.051), while initial chemotherapy was not associated with local recurrence (sHR 1.03, 95% CI 0.60–1.76; p = 0.910).

**Table 5.** Fine–Gray Competing-Risk Regression for Local Recurrence. Sub-distribution Hazard Ratios (sHR) With 95% Confidence Intervals are Shown on a Logarithmic Scale

Variable	sHR (95% CI)	p value
Intralesional margin	2.08 (1.20–3.63)	0.010
Neurofibromatosis type 1	1.64 (1.04–2.61)	0.034
Metastases at presentation	1.85 (0.72–4.78)	0.200
Initial radiotherapy	0.62 (0.38–1.00)	0.051
Initial chemotherapy	1.03 (0.60–1.76)	0.910

Abbreviations: sHR, sub-distribution hazard ratio; CI, confidence interval; NF1, neurofibromatosis type 1.

sHR, sub-distribution hazard ratio; CI, confidence interval.

## Discussion

This meta-analysis synthesized the largest series of published spinal and paraspinal MPNST cases to date and identified key prognostic factors. High early mortality and local recurrence risk highlight the aggressive nature of the disease. Metastatic stage at presentation and MTT histology signaled poor survival. GTR emerged as the dominant surgical predictor of OS, while margin status influenced local recurrence risk.

The patient profile in this study resembled that of previous MPNST reports with a narrow male predominance, a one-third association with NF1, and a one-fifth association with a history of irradiation.^6,136-138^ While the lifetime risk of MPNST has been reported to be increased by more than 1000-fold in association with NF1^139^, and tumors arise at a younger age, reports conflict as to whether NF1 status affects prognosis. Ducatman et al. reported impaired OS with NF1 ^10^, while Doorn et al. associated NF1 status with an increased risk of a second MPNST occurrence.^140^ Chou et al. explained that MPNSTs oftentimes arise in plexiform neurofibroma bundles, complicating total removal and conveying the continued growth secondary to NF1 rather than MPNST.^6^ Belakhoua et al. described low-grade MPNSTs – a minority among MPNSTs – as often associated with NF1.^1^ A recent multinational study did not observe survival differences between NF1-associated and sporadic MPNST cases.^9^ In our data, NF1 status was not associated with OS. An increased risk of local recurrence with NF1 was demonstrated in a competing-risk analysis. Data on tumor grade were too limited to be included in the analysis.

MTT histology, a rare subset of MPNST containing rhabdomyoblastic differentiation, accounts for 5–10% of MPNST cases and entails a clearly formidable prognosis.^46^ The percentage of MTT cases in the present data (7.4%) with remarkably high early mortality corroborates previous findings. Published expert opinions recommend aggressive resections whenever achievable, although their efficacy has not been clearly demonstrated in this subpopulation.^11,23,141^

MPNSTs in this series were evenly distributed across the spinal axis. Prognosis trended to improve toward more caudal locations. This may be related to more permissive conditions for adequate resection in the lumbo-sacral area. Intramedullary tumor location suggested inferior survival compared with other morphologies, likely attributable to insufficient resectability inside the spinal cord. Previous studies have not compared prognoses across anatomic and morphologic subtypes.

This study, the largest series of spinal and paraspinal MPNST tumors to date, demonstrated macroscopic total resection (GTR) as the key treatment-related predictor of OS. Previous studies have been ambivalent about the role of GTR in OS, likely owing to low sample sizes given the rarity of axial MPNSTs. Kolber et al. combining Norwegian, Swedish, and Italian populations, demonstrated survival benefits secondary to GTR across the entire cohort, but not in the smaller subpopulation with NF1.^9^ A study using the Surveillance, Epidemiology, and End Results (SEER) database covering 26% of the United States population indicated higher survival rates after GTR when compared with STR or biopsy alone.^3^ Irrespective of the extent of resection, a substantial percentage of MPNST patients suffer from high early mortality. Metastatic stage at diagnosis and MTT histology have been widely attributed to early mortality,^9,46,136^ which the present study echoes.

In extremity MPNSTs, *en bloc* resections with clear, wide margins have been demonstrated to raise otherwise inferior survival to the level of other soft-tissue sarcomas.^142^ In axial locations, the vicinity of critical structures, including the dura mater, spinal cord and nerves, great vessels, and esophagus, usually precludes attainment of wide margins, and removal through an intact tumor pseudocapsule is pursued.^143^ Following GTR with a microscopically positive resection surface or piecemeal resection, microscopic residual tumor is expected. In their single-institution MPNST study, Anghileri et al. reported a 2.43-fold increased local recurrence risk with positive resection marginals.^144^ Among 23 NF1-associated MPNSTs, Dunn et al. demonstrated 2.3-fold improved overall and local survival following margin-negative resection.^5^ These findings align with our results of improved OS and LRFS with negative margin resections. Overall, MPNSTs tend to recur locally rather than distantly, with the highest recurrence rate among soft tissue sarcomas at 40 to 70%.^138^

Chou et al. reported the largest multicenter series of spinal MPNSTs to date, in which they could not demonstrate that Enneking-appropriate resection (*en bloc* resection with macroscopically and microscopically intact margins) produced overall or local survival benefit.^6^ This was partially attributed to the still low number of subjects (n = 27) and partially to the overall poor prognosis of axial MPNSTs regardless of the type of resection. This underscores the importance of the present findings on the role of margin status in local recurrence risk with axial MPNST. While margin status added limited prognostic value for OS beyond macroscopic resection completeness, competing-risk analysis demonstrated that intralesional surgeries result in increased local recurrence risk among those surviving long enough to experience local failure. This highlights margin status as a secondary but clinically meaningful determinant of treatment outcome.

Many authors advocate adjuvant radiotherapy to improve local control despite ambiguous results on its efficacy.^6^ In the SEER database analysis, radiotherapy was associated with inferior outcomes,^3^ while several studies have observed no survival effects secondary to radiotherapy.^6,9,136^ Interpretation of these findings is limited by the retrospective nature of the data, as radiotherapy is more likely to be administered in patients with more aggressive or residual disease, which introduces confounding by indication. In the present study, radiotherapy was associated with improved OS, whereas its effect on local recurrence was borderline and not clearly protective in a competing-risk analysis of local recurrence. These findings likely reflect treatment selection bias rather than a causal effect and underscore the uncertainty surrounding the role of radiotherapy in spinal MPNST.

In summary, these results support the consideration of macroscopic total resection when anatomically feasible, with margin optimization most relevant for local control. Substantial early mortality and local recurrence risk necessitate careful discussion of treatment decisions with the patient. Initial systemic staging is imperative owing to the dismal prognosis associated with metastatic disease. Early and vigilant postoperative surveillance is essential given the high early local recurrence risk.

### Limitations

This study is limited by its retrospective, literature-based design and reliance on reported cases and small series. The high potential for selection and publication bias render the present findings hypothesis generating rather than conclusive. The very high number of screened abstracts and the single-screener approach may have also increased selection bias. Important variables, such as tumor size, grade, and standardized margin definitions, were inconsistently reported, limiting adjustment for additional confounders. Treatment allocations were nonrandomized, and associations with radiotherapy and chemotherapy were subject to confounding by indication. Interaction between extent of resection and radiotherapy could not be reliably assessed because of limited event numbers.

Despite representing the largest spinal MPNST cohort reported, event numbers were limited, and multivariable analyses should be interpreted cautiously. Regardless, consistency across Cox and competing-risk models strengthens confidence in the main conclusions.

## Conclusions

Spinal and paraspinal MPNSTs are characterized by poor survival and high early local recurrence risk. Extent of resection is the dominant treatment-related determinant of OS, whereas margin status primarily influences local recurrence risk. These findings support the consideration of macroscopic total resection when anatomically feasible, even when negative margins cannot be achieved, although this should be interpreted as hypothesis generating. Negative margins may be most relevant for local disease control after GTR.

## Supplemental material

Supplemental material - Spinal and Paraspinal Malignant Peripheral Nerve Sheath Tumors (MPNSTs): Survival, Local Recurrence, and the Relative Importance of Resection Extent and Margin Status Supplemental material for Spinal and Paraspinal Malignant Peripheral Nerve Sheath Tumors (MPNSTs): Survival, Local Recurrence, and the Relative Importance of Resection Extent and Margin Status by Leevi Toivonen, Anton Denisov in Global Spine Journal

Supplemental material - Spinal and Paraspinal Malignant Peripheral Nerve Sheath Tumors (MPNSTs): Survival, Local Recurrence, and the Relative Importance of Resection Extent and Margin Status Supplemental material for Spinal and Paraspinal Malignant Peripheral Nerve Sheath Tumors (MPNSTs): Survival, Local Recurrence, and the Relative Importance of Resection Extent and Margin Status by Leevi Toivonen, Anton Denisov in Global Spine Journal

## Data Availability

The study data are available in the manuscript and data supplements.*
