# Supplementary material for: Spinal and Paraspinal Malignant Peripheral Nerve Sheath Tumors (MPNSTs): Survival, Local Recurrence, and the Relative Importance of Resection Extent and Margin Status
Source: Global Spine J. 2026 Jul 2:21925682261465343. Online ahead of print. doi: 10.1177/21925682261465343 (PMC13328113; doi:10.1177/21925682261465343)
Supplement: Supplemental material - Spinal and Paraspinal Malignant Peripheral Nerve Sheath Tumors (MPNSTs): Survival, Local Recurrence, and the Relative Importance of Resection Extent and Margin Status [file sj-pdf-2-gsj-10.1177_21925682261465343.pdf]

Supplementary Material

Supplementary Figure S1

OS by margin

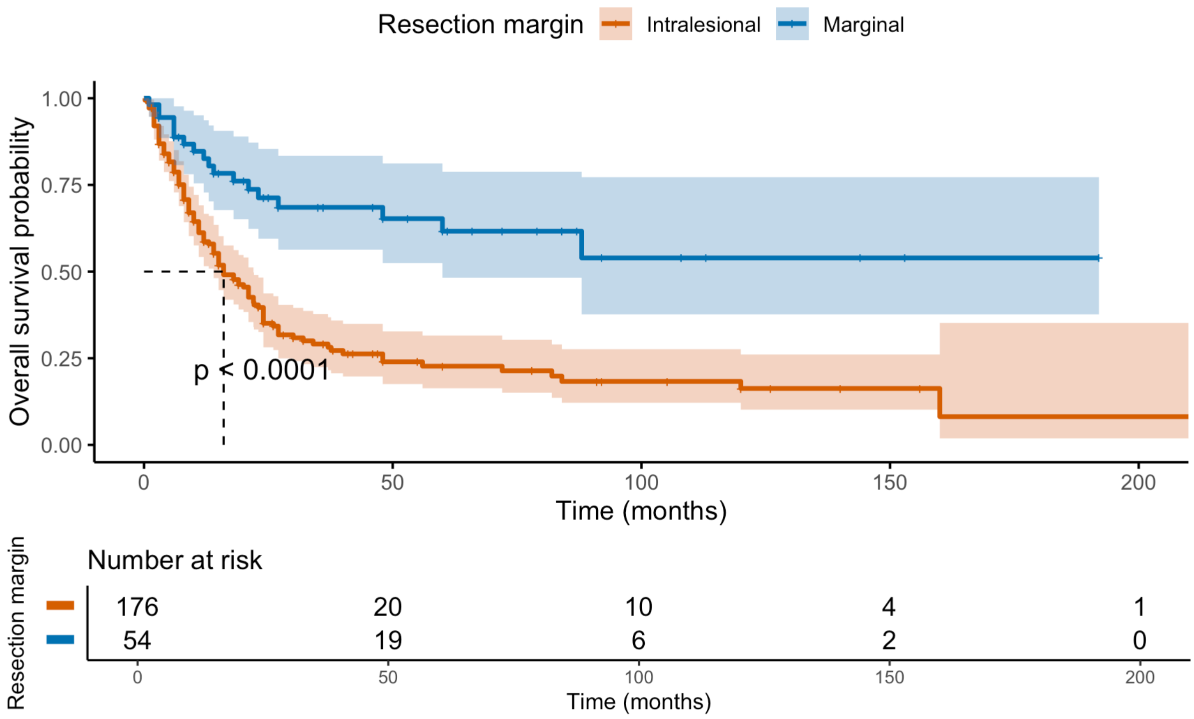

Figure S1. Estimated overall survival (OS) stratified by resection margin status. Patients who underwent marginal resection demonstrated significantly improved survival compared with those with intralesional margins (log-rank $p < 0.0001$).

Supplementary Figure S2

LRFS overall cohort

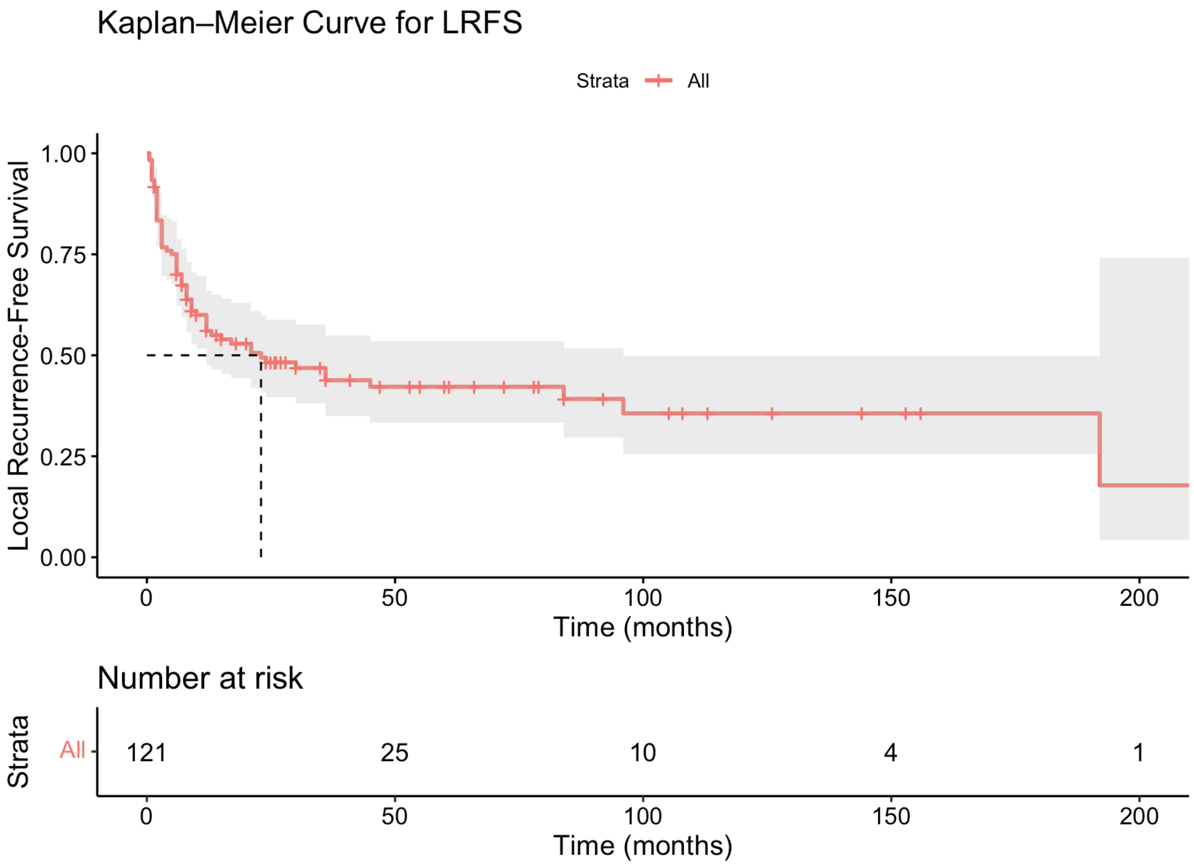

Figure S2: Estimated local recurrence free survival (LRFS) for the whole cohort. Shaded areas represent the 95% confidence intervals, and tick marks indicate censored observations. The number of patients at risk at predefined timepoints is presented below the plot.

Table S1. Comparison of Cox regression models for overall survival using AIC stratified by histology type

Model	Variables Included	df	AIC	ΔAIC vs Best
Model 1 (Extent model)	Age, metastases at diagnosis, NF1 status, extent of resection (GTR vs STR), initial radiotherapy, initial chemotherapy	6	987.6	0
Model 2 (Margin model)	Age, metastases at diagnosis, NF1 status, surgical margin, initial radiotherapy, initial chemotherapy	6	995.2	+7.6

Lower AIC values indicate better model fit. The model including extent of resection (Model 1) demonstrated the best performance. The combined model provided no meaningful improvement, while the margin-only model showed inferior fit, supporting extent of resection as the dominant surgical predictor of overall survival.

Table S2. Distribution of Surgical Margin by Extent of Resection

Margin Status	GTR, n (%)	STR, n (%)	Total (n)
Intralesional	44 (60.3%)	29 (39.7%)	73
Marginal	48 (100%)	0 (0%)	48
Total	92	29	121

Percentages are calculated within margin categories (row percentages).

GTR, gross total resection; STR, subtotal resection

Table S3. Comparison of Cox regression models for local recurrence–free survival (LRFS) using AIC

Model	Variables Included	df	AIC	ΔAIC vs Best
Model 1 (Extent model)	Age, histology, initial radiotherapy, initial chemotherapy, extent of resection (STR vs GTR)	5	398.3	0
Model 2 (Margin model)	Age, histology, surgical margin, initial radiotherapy, initial chemotherapy	6	403.6	+5.3

Lower AIC values indicate better model fit. The model including extent of resection (Model 1) demonstrated the best performance. The combined model provided no meaningful improvement, while the margin-only model showed inferior fit, supporting extent of resection as the dominant surgical predictor of local recurrence survival.

Table S4: Estimated median survival based on anatomic location and tumor morphology

Group	Estimated median OS	95% CI	Estimated median PFS	95% CI
**Anatomic region**				
Cervical	15	(12.0–19.0)	8.0	(6.0–12.0)
Thoracic	17	(14.0–21.0)	10.0	(8.0–13.0)
Lumbar	20	(16.0–26.0)	12.0	(9.0–18.0)
Sacral	36	(24.0–60.0)	25.0	(18.0–36.0)
Multiple	6	(4.0–9.0)	3.0	(2.0–5.0)
N/A	14	(10.0–19.0)	7.0	(5.0–11.0)
**Tumor morphology**				
Intramedullary	8.0	(5.0–15.0)	4.0	(2.0–8.0)
Intradural	19.0	(16.0–23.0)	12.0	9.0–16.0)
Extradural-intraspinal	16.0	(12.0–21.0)	9.0	(6.0–14.0)
Dumbbell	15.0	(12.0–19.0)	8.0	(6.0–12.0)
Intraosseous	14.0	(10.0–22.0)	7.0	(5.0–12.0)
Paraspinal	16.0	(13.0–21.0)	10.0	(7.0–14.0)
N/A	14.0	(11.0–19.0)	7.0	(5.0–11.0)

OS, overall survival; RFS, recurrence free survival; CI, confidence interval; N/A, not available
